# Association Analysis between *SPP1*, *POFUT1* and *PRLR* Gene Variation and Milk Yield, Composition and Coagulation Traits in Sarda Sheep

**DOI:** 10.3390/ani10071216

**Published:** 2020-07-17

**Authors:** Maria Luisa Dettori, Michele Pazzola, Elena Petretto, Giuseppe Massimo Vacca

**Affiliations:** Department of Veterinary Medicine, University of Sassari, via Vienna 2, 07100 Sassari, Italy; mldettori@uniss.it (M.L.D.); elenapetretto@outlook.it (E.P.); gmvacca@uniss.it (G.M.V.)

**Keywords:** sheep milk, coagulation traits, *SPP1*, *POFUT1*, *PRLR*

## Abstract

**Simple Summary:**

The purpose of our research was to analyze the association between the three candidate genes secreted phosphoprotein 1 (*SPP1*), protein O-fucosyltransferase 1 (*POFUT1*) and prolactin receptor (*PRLR*) with milk production, quality and coagulation properties in 380 Sarda dairy sheep. Results revealed an association between *SPP1* and somatic cells count, in line with the function of this gene and with its genomic position. We revealed an association of *POFUT1* variation with milk coagulation properties, and *PRLR* with quality. This information can be useful for future breeding schemes in sheep.

**Abstract:**

Many studies focus on the identification of genomic regions that undergo selective processes, where evidence of selection is revealed and positional candidate genes are identified. The aim of the research was to evaluate the association between positional candidate genes, namely secreted phosphoprotein 1 (*SPP1*, sheep chromosome *Ovis aries* OAR6, 36.651–36.658 Mb), protein O-fucosyltransferase 1 (*POFUT1*, OAR13, 61.006–61.027 Mb) and prolactin receptor (*PRLR*, OAR16, 38.969–39.028 Mb) with milk yield, composition and coagulation traits. Eight single nucleotide polymorphisms (SNPs) mapping to the three genes were genotyped in 380 Sarda dairy sheep. Statistical analysis revealed an association between SNP rs161844011 at *SPP1* (chromosome position Oar_v3 OAR6:36651870, gene region exon 7) and somatic cell score, while *POFUT1* SNP rs424501869 (OAR13:61007495, intron 1) was associated with curd firmness both 45 and 60 min after rennet addition (*p* = 0.015 and *p* = 0.007, respectively). SNP rs400874750 at *PRLR* gene (OAR16:39004070, intron 2) had a significant association with lactose content (*p* = 0.020), somatic cell score (*p* = 0.038), rennet coagulation time (*p* = 0.018) and curd firming time (*p* = 0.047). The outcome of this research confirmed predictions based on genomic studies, producing new information regarding the *SPP1*, *POFUT1* and *PRLR* genes, which may be useful for future breeding schemes.

## 1. Introduction

In the last decades, the increase of sheep milk and cheese production has been recorded worldwide [1]. The enhancement of cheese and other dairy products is often pursued by official labelling, such as in European Union countries where protected designation of origin (PDO) and protected geographical indication (PGI) marks have a significant positive impact on the economic value of products [2]. Nevertheless, the starting point for the improvement of dairy products is based on the study of milk quality [3]. Phenotypic traits of milk, i.e., fat and protein content, have a key role in influencing cheese yield and the quality of dairy products, and several studies have also evidenced the effect of genotype on milk composition and coagulation traits in sheep [4]. Many genes are considered as functional candidates because of their impact on a biological function and then on a specific trait. The casein genes, clustered on chromosome 6 in cattle, goat and sheep, were among the first candidate genes for dairy traits examined [5,6,7]. The *GH* (growth hormone) and *GHR* (growth hormone receptor) genes have been investigated as candidate genes due to their metabolic function [8,9], and the myostatin (*GDF8*) gene for its effect on a biosynthetic pathway [10]. More recently, based on genome-wide studies, it has been possible to identify genomic regions that have undergone recent selective processes, such as selective sweeps, which allowed for the detection of positional candidate genes. Gutierrez-Gil et al. [11] compared whole genomes of dairy and non-dairy sheep breeds and identified several positional candidate genes, including, among others, secreted phosphoprotein 1 (*SPP1*), protein O-fucosyltransferase 1 (*POFUT1*) and prolactin receptor (*PRLR*). 

The *SPP1* is a 6.55 kb long gene, with seven exons, encoding a protein of 279 amino acid residues (https://www.ensembl.org/index.html). *SPP1* is located in the first half of the *Ovis aries* chromosome OAR6 (36.651–36.658 Mb), a region of positive selection for milk traits orthologous to a region on BTA6 (*Bos Taurus* chromosome 6) where many quantitative trait loci (QTLs) for milk traits have been identified in cattle [11]. The protein encoded by *SPP1* is also known as osteopontin (OPN) because of its role in the activities of fibroblasts, osteoblasts and osteocytes in promoting bone tissue growth [12]. Successive studies about osteopontin have evidenced its functions in the binding of cell surface and embryonal implantation to establish a functional placenta during the initial stages of pregnancy in many mammalian species, and its secretion by the macrophages to improve cell-mediated immunity [13,14]. A variation at *SPP1* was reported to regulate milk protein gene expression [15], mastitis resistance [16] and lactation persistency in dairy cows [17]. 

*POFUT1* is a 20.41 kb long gene, and it includes 7 exons encoding a protein of 391 amino acid residues known as O-fucosyltransferase (https://www.ensembl.org/index.html). *POFUT1* is located on chromosome OAR13 (61.006–61.027 Mb) in a candidate region including genes involved in mammary development and differentiation and mastitis defense [11]. O-fucosyltransferase is an enzyme located in the lumen of the endoplasmic reticulum, responsible for the addition of fucose to epidermal growth factor-like (EGF-like) repeats (O-fucosylation) in many molecules, such as glycans, glycoproteins and glycosphingolipids [18]. The POFUT1 protein has two N-glycosylation sites, the first of which, N65, is highly conserved in bilaterians and is critical for protein functionality [18]. O-fucosylation also occurs in the Notch receptors; transmembrane receptor activation by the Notch ligands regulate cell fate in metazoans, and the Notch receptors without O-fucose are inactive [19].

The sheep *PRLR* is a 58.85 kb long gene with 9 exons coding for a protein of 581 amino acid residues (https://www.ensembl.org/index.html). *PRLR* was mapped to OAR16 (38.969–39.028 Mb), a region where positive selection was reported after haplotype analysis in sheep [20]. *PRLR* is a membrane-anchored protein identified in many tissues of adult mammals, classified in the class 1 cytokine receptor superfamily and in the same family of growth hormone receptor [21]. Two distinct isoforms of the PRL receptor, namely long and short, are produced by alternative splicing [22], and their differential expression varies as a function of the different stages of reproduction and lactation [21]. The polymorphism of *PRLR* has been associated with maternal behavior [23] and with changes of the mammary gland throughout lactation [24].

The association of these three positional candidate genes with milk traits have scarcely been studied in sheep. The aim of the present study was to investigate the association between *SPP1*, *POFUT1* and *PRLR* and milk yield, composition and coagulation traits in dairy sheep.

## 2. Materials and Methods 

### 2.1. Ethics Approval

No specific authorization from an animal ethics committee was required. Blood samples for DNA isolation and milk samples were collected by two of the authors (M.P. and G.M.V.), who are experienced veterinarians, concurrently with official performance controls of the flock book, which were not directly linked with the present trial. Sheep were included in this trial with the agreement of the farmers on a voluntary basis.

### 2.2. Animals, Farms and Sampling

A population of 380 ewes from 19 farms (20 ewes at each farm) located in Sardinia (Italy) was used for the study. A detailed description of the farms, animals and sampling procedures are reported in Pazzola et al. [25] and Vacca et al. [26]. In brief, all the ewes and farms were officially registered in the flock book of the Sarda sheep breed; farms were mainly managed with semi-extensive methods; multiparous sheep sampled for the present study lambed in November–December, whereas primiparous sheep lambed in the successive February–March, as normally happens in semi-extensive sheep farming in Sardinia. Ewes were between the first and ninth parity, and two to seven months after lambing. Individual milk samples were taken at each farm in a single day (one day sampling for each farm) throughout a single lactation period from spring to summer in 2012. During the afternoon milking, milk samples were collected in 200 mL sterile plastic containers and kept at 4 °C until analyses. Daily milk yield was recorded as the morning plus evening milking of the same sampling date. 

A blood sample was taken from each ewe in K_3_EDTA vacuum tubes (BD Vacutainer, Becton Dickinson, Franklin Lakes, NJ, USA) for DNA extraction.

### 2.3. Milk Analysis

Milk samples were analyzed within 24 h after collection for composition and milk coagulation properties. Fat, protein, casein, lactose content and pH were measured using a MilkoScan FT6000 device (Foss Electric A/S, Hillerød, Denmark), according to the International Organization for Standardization and International Dairy Federation (ISO-IDF) standard [27]. Daily fat and protein yield in g/day (dFPY) was calculated as the sum of fat protein content multiplied by daily milk yield. Total bacterial count (TBC) was measured using a BactoScan FC150 instrument (Foss Electric) according to the ISO-IDF standard [28] and later, in order to normalize the distribution, transformed into the log-bacterial count (LBC = log_10_ (total bacterial count/1000)), according to the ISO-IDF standard [29]. Somatic cell count (SCC) was measured using a Fossomatic 5000 equipment (Foss Electric) according to the ISO-IDF method [30] and transformed into the somatic cell score (SCS = log_2_ (SCC × 10^−5^) + 3) according to Shook [31]. Milk coagulation properties (MCPs) were measured using a Formagraph instrument (Foss Italia, Padova, Italy) and the method first described in McMahon and Brown [32] and modification reported for the sheep species in Pazzola et al. [25]. Briefly, a volume of 10 mL of each milk sample was heated to 35 °C and mixed with rennet enzyme (200 μL of a solution with final milk clotting units (IMCUs) per mL of 0.0513/mL of milk, obtained with the dilution in distilled water 1.2% (wt/vol) of Hansen Naturen Plus 215 (Pacovis Amrein AG, Bern, Switzerland; 80 ± 5% chymosin and 20 ± 5% pepsin; 215 international milk clotting units per mL, IMCU/mL)) and analyzed for 60 min after rennet addition. The following MCPs were measured: RCT (rennet coagulation time in min); k_20_ (curd firming time in min); and a_30_, a_45_ and a_60_ (curd firmness 30, 45 and 60 min after rennet addition, in mm). Curd firmness over time (CF_t_) traits were calculated using the records of curd firmness downloaded from the Formagraph and the method first described in Bittante [33] and modification reported for the sheep species in Vacca et al. [26]. The following CF_t_ were measured: RCT_eq_ (rennet coagulation time estimated by the CF_t_ equation, in min); CF_P_ (the maximum potential curd firmness at an infinite time, in mm); k_CF_ (curd-firming rate constant, in % × min^−1^); k_SR_ (syneresis rate constant, in % × min^−1^); CF_max_ (maximum curd firmness, in mm); and t_max_ (time to attain CF_max_, in min).

### 2.4. DNA and Haplotype Analyses

Genomic DNA was extracted using the Gentra Puregene blood kit (Qiagen, Hilden, Germany), and purity and concentration were measured with an Eppendorf BioPhotometer (Eppendorf, Hamburg, Germany). A custom open array, based on the TaqMan real-time PCR assay, was designed for single nucleotide polymorphism (SNP) genotyping. It included a total of 8 SNPs: 2 from the *SPP1* gene, 3 from *POFUT1* and 3 from *PRLR* (Table 1). Context sequences are given in Supplementary Information Table S1. Genotyping was carried out using a 12 K Flex QuantStudio instrument (Thermo Fisher Scientific, Waltham, MA, USA). Genotypes were visualized with Taqman Genotyper v.1.3 software (Applied Biosystems, Waltham, MA, USA). 

The Haploview software package [34] was used to estimate and plot pairwise linkage disequilibrium (LD) measures (*D*′ and r2) and to infer haplotype frequencies as well as to define LD blocks according to the Gabriel criteria [35]. Haploview was also used to estimate minor allele frequencies (MAF) and observed and expected heterozygosites and to identify significant departures from the Hardy–Weinberg equilibrium at each polymorphic locus.

### 2.5. Statistical Analyses

Association analysis between the genotypes of the polymorphic SNPs at *SPP1*, *POFUT1* and *PRLR*, with milk composition, MCP and CF_t_ traits was performed using the MIXED procedure of SAS (version 9.4, SAS Inst. Inc., Cary, NC, USA) and the following model (1):

Y_ijklm_ = µ + G_i_ + F_j_ + P_k_ + DIM_l_ + SIRE(G)_m_ + e_ijklm_ (1)

where Y_ijklm_ is the observed trait of milk composition, MCP and CF_t_; µ is the general mean; G_i_ is the fixed effect of the i^th^ SNP genotype (i = 2 to 3 levels: the two homozygotes and the heterozygote); F_j_ is the fixed effect of the j^th^ farm, which also includes animal management and feeding (j = 1 to 19 levels; i.e., the different farms where animals were reared); P_k_ is the fixed effect of k^th^ parity of the ewes (k = 1 to 4 levels; first to fourth or more parities); DIM_l_ is the fixed effect of the l^th^ days in milking (l = 4 levels; level 1: ≤100 days; 2: 101–140 days; 3: 141–160 days; level 4: ≥161 days); SIRE(G)_m_ is the random effect of the m^th^ sire (m = 108 different sires) nested within the genotype; and e_ijklm_ is the error random residual effect. We analyzed one milk trait for each SNP at a time. We only considered SNPs with MAF > 0.05. 

In order to investigate the association between milk traits and each of the LD blocks, the same model (1) was slightly modified into model (2), with LD_i_ (i = 3 levels) instead of G_i_, one milk trait for each LD block at a time. Correction for multiple testing for both models (1) and (2) were performed using the Bonferroni method at α = 0.05.

## 3. Results 

Descriptive statistics regarding all the milk traits recorded from the sampled population of 380 Sarda ewes are summarized in Supplementary Information Table S2. Values of curd firmness at 0 mm, both for MCP and CF_t_, were labelled as missing and were not computed in the statistical analysis (a_30_: n = 3; a_45_: n = 2; a_60_: n = 3; CF_P_: n = 7; CF_max_: n = 4).

### 3.1. Allele Frequencies at SPP1, POFUT1 and PRLR, and Association Analysis with Milk Traits 

The SNPs analyzed in the present experiment were chosen taking into account the technical requirements of a TaqMan^®^ assay, and by using the Ensembl Genome Browser (https://www.ensembl.org/index.html) to localize and choose each SNP. For the *SPP1* gene we analyzed one coding SNP (rs161844011) causing the amino acid substitution p.Gln235Arg, and one exonic SNP (rs426249393) in the 5’ untranslated region (UTR); the other SNPs analyzed were intronic. Among the eight SNPs genotyped, only rs421284407 (*POFUT1* intron 2) was monomorphic; all the others were polymorphic, with minor allele frequencies (MAF) higher than 0.1. The population analyzed was in Hardy–Weinberg equilibrium at all loci (Table 1).

Results of the statistical analysis (*F*-values and significance) from model (1), performed to investigate the influence between each of the seven polymorphic SNPs and milk traits, are reported in Supplementary Information Table S3. The results regarding the fixed effects of farm, parity and stage of lactation included in model (1) showed high levels of significance for almost all the milk traits (data not reported in tables), as already discussed in previous papers using datasets connected to that of the present study [7,25]. 

**Figure 1:**
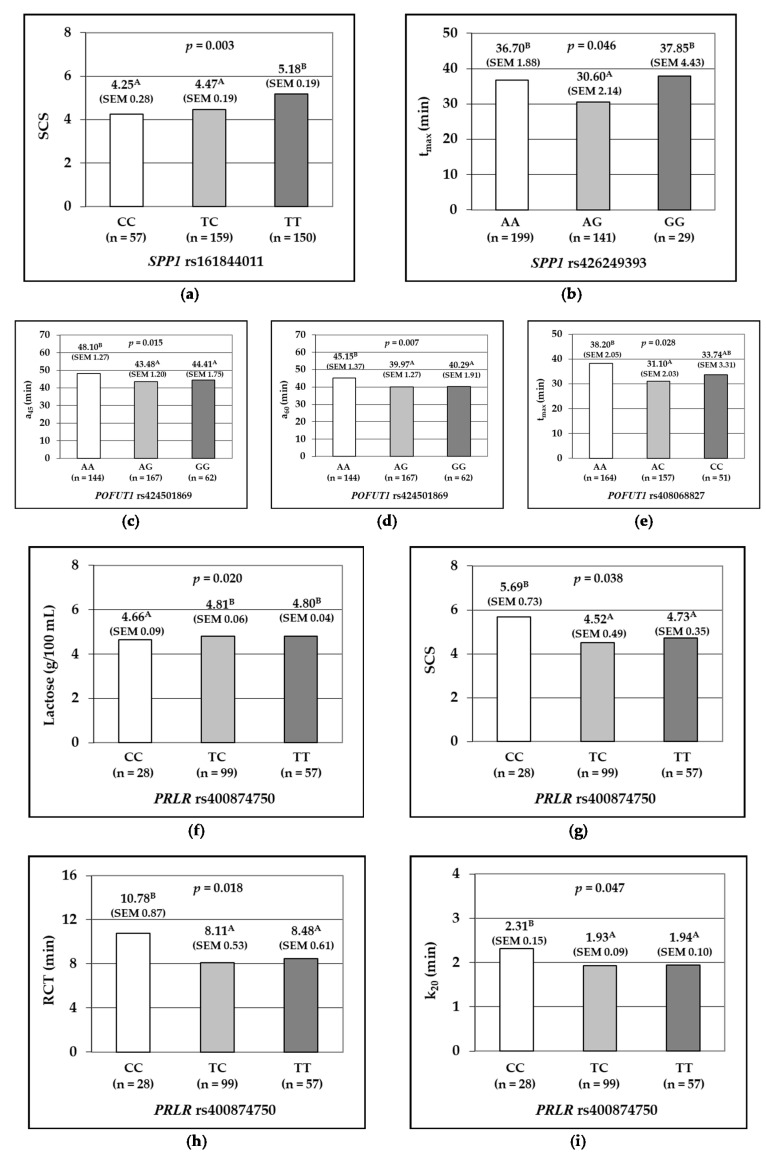
*p*-Values, least squares means and standard errors of the means (SEM, between brackets) of milk traits according to the genotypes at the single SNP. Gene *SPP1*: the association of rs161844011 with somatic cell score, SCS (**a**); rs426249393 with time to attain maximum curd firmness, t_max_ (**b**). Gene *POFUT1*: rs424501869 with curd firmness 45 min after rennet addition, a_45_ (**c**) and 60 min, a_60_ (**d**); rs408068827 with time to attain the maximum curd firmness, t_max_ (**e**). Gene *PRLR*: rs400874750 with lactose (**f**), SCS (**g**), rennet coagulation time, RCT (**h**) and curd firming time, k_20_ (**i**). ^A,B^ Different capital letters on the right of least squares means in the same graphic differ significantly.

In the *SPP1* gene, the rs161844011 SNP marker was associated with SCS (*p* = 0.003), and ewes carrying the homozygote TT genotype displayed the highest value (Figure 1a). The *SPP1* SNP rs426249393 was associated with t_max_ (*p* = 0.046), and homozygous ewes AA and GG showed delayed times to attain the maximum curd firmness, about six to seven minutes later than heterozygous AG (Figure 1b).

The SNPs investigated at *POFUT1* were associated with curd firmness traits. Milk samples from rs424501869 AA ewes were characterized by the highest value of a_45_ (48.10 min; *p* = 0.015) and a_60_ (45.15 min; *p* = 0.007) (Figure 1c,d), whereas milk samples from homozygous rs408068827 AA ewes were associated with the longest time to attain the maximum curd firmness, 38.20 min (*p* = 0.028; Figure 1e).

The SNP rs400874750 at *PRLR* gene was significantly associated with milk composition and coagulation times: milk from CC homozygous ewes displayed the lowest lactose concentration at 4.66 mg/100 mL (*p* = 0.020; Figure 1f) and the highest SCS at 5.69 (*p* = 0.038; Figure 1g). In addition, milk produced by rs400874750 CC ewes had delayed times both for RCT (*p* = 0.018) of about 2.5 min of delay in comparison with TC and TT, and k_20_ (*p* = 0.047), about 0.30 min delay (Figure 1h,i).

### 3.2. Linkage Disequilibrium and Association Analysis between Haplotype Blocks and Milk Traits

**Figure 2:**
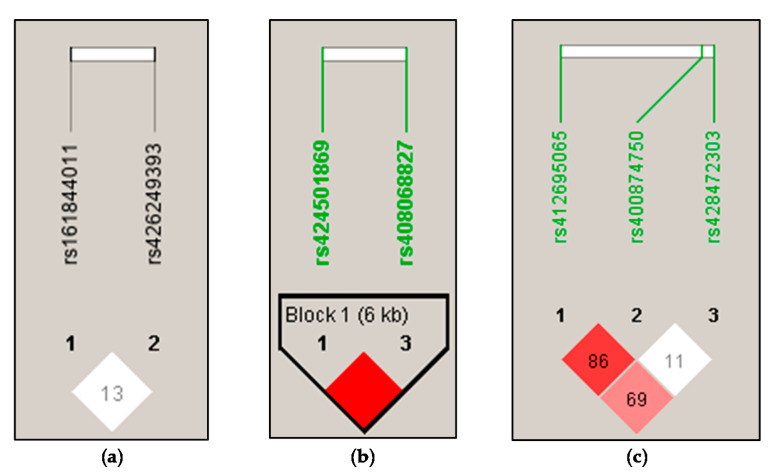
Haplotype blocks defined by the SNPs at *SPP1*, *POFUT1* and *PRLR* genes in Sarda sheep (n = 380). (**a**) Linkage disequilibrium structure of SNPs at the *SPP1* gene. SNPs: 1, rs161844011; 2, rs426249393. (**b**). Linkage disequilibrium structure and Block 1 of SNPs at the *POFUT1* gene. SNPs: 1, rs424501869; 3, rs408068827. (**c**) Linkage disequilibrium structure of SNPs at the *PRLR* gene. SNPs: 1, rs412695065; 2 rs400874750; 3 rs428472303. Haploview plot of pairwise *D*′: red, *D*′ = 1.0 and logarithm of the odds (LOD) ≥ 2.0; white, *D*′ < 1.0 and LOD < 2.0. LD blocks are delimited by a black line. Haplotype tagging SNPs are within black boxes. SNPs in bold are haplotype tagging SNPs (htSNP).

The LD analysis performed on the three genes revealed only one LD block (Figure 2). The haplotype tagging SNPs were evidenced at the *POFUT1* gene, rs424501869 and rs408068827, which were enclosed in Block 1 (Figure 2b) showing three haplotypes: AA (frequency 0.610); GA, (frequency 0.042); GC (frequency 0.348).

Haplotypes at Block 1 and milk traits were submitted to statistical analysis by using model (2). Results of the statistical analysis (*F*-values and significance) are reported in Supplementary Information Table S4. Similarly to the results regarding the investigation of single SNPs, fixed effects of farm, parity and stage of lactation included in model (2) showed high levels of significance, but these are not reported in tables.

**Figure 3:**
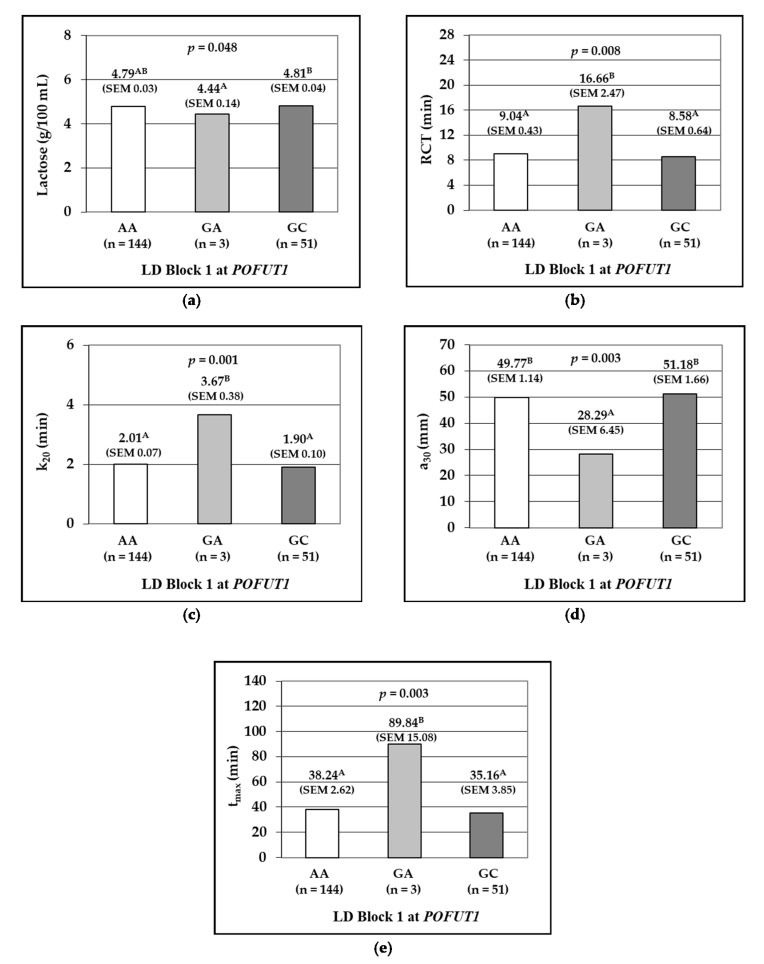
*p*-Values, least squares means and standard errors of the means (SEM, between brackets) of milk traits according to the haplotypes at Block 1 of SNPs at the *POFUT1* gene. The association of Block1 with lactose (**a**); rennet coagulation time, RCT (**b**); curd firming time, k_20_ (**c**); curd firmness 30 min after rennet addition, a_30_ (**d**); and time to attain maximum curd firmness, t_max_. (**e**). ^A,B^ Different capital letters on the right of least squares means in the same graphic differ significantly.

Haplotypes at Block 1 were significantly associated with milk lactose content (*p* = 0.048), with the lowest concentration for GA ewes (Figure 3a). Regarding RCT and k_20_, milk samples from ewes carrying the GA haplotypes showed rennet coagulation time about 7 min (*p* = 0.008; Figure 3b), and curd firming time about 1.70 min (*p* = 0.001; Figure 3c) longer than AA and GC. Finally, in GA ewes curd firmness measured by a_30_ was smaller (*p* = 0.003; Figure 3d) and t_max_ delayed (*p* = 0.003; Figure 3e).

## 4. Discussion

We evaluated the association between *SPP1*, *POFUT1* and *PRLR* genes and milk production, composition and coagulation properties in 380 Sarda breed ewes. We considered the abovementioned genes because they had been reported to be positional candidates in relation to specific dairy traits [11], and they have been poorly studied for their effects in many sheep populations. 

Osteopontin (encoded by the *SPP1* gene) is involved in immune regulation and tissue remodeling [36], and it plays an important role in mammary gland development and local immunity, as well as in milk production [16]. Gutierrez-Gil et al. [11] found that *SPP1* is located in a genomic region on OAR6, a candidate for milk production traits and lactation regulation in sheep. Ruiz-Larrañaga et al. [37] reported that *SPP1* is in a highly selected genomic region displaying selection signatures in Latxa sheep, a breed which has undergone long dairy selection pressure. Yurchenko et al. [38] performed high-density genotyping of 15 local sheep breeds from Russia and found that *SPP1* is in a candidate region for milk and lactation traits. However, other studies carried out in cattle and sheep populations are not in accordance with that last hypothesis. The *SPP1* SNP c.8514C>T was investigated in Holstein–Friesian cattle [39] and in the dairy cattle breed Girolando [40], and no significant associations with disease resistance or milk parameters were evidenced. García-Fernández et al. [41] did not find any significant association between *SPP1* polymorphisms and milk production traits in a commercial population of Churra ewes.

In the present study, one exonic SNP of the *SPP1* gene, rs161844011, was significantly associated with SCS, with the TT genotype displaying the highest least mean square. The SNP rs161844011 is a missense variant located on exon 7, causing the amino acid variation p.Gln235Arg, which has a score of 0.24 according to the SIFT (scale-invariant feature transform) algorithm (http://sift.jcvi.org/), indicating it is tolerated by the protein, based on sequence homology. The influence of the *SPP1* SNP rs161844011 on SCS confirmed predictions based on genomic studies, searching for regions under selection. Alain et al. [16] studied three SNPs in the promoter region and one SNP in the 3’UTR of *SPP1* and evidenced the occurrence of a link with SCS and resistance to mastitis in dairy cows. They also found evidence of the presence of haplotype blocks among the different SNPs, which were not evidenced in the present study (Figure 2a). Even if in sheep SCC is still far from being accepted as a universal tool to reveal mastitis because of the different cut-off limits based on the management or the breed [42], it is known that in the Sarda breed appropriate values of heritability can be exploitable to improve somatic cells values in breeding schemes at the field level [43]. In addition, the identification of novel favorable *SPP1* genotypes in sheep is a good perspective to investigate the key role of osteopontin in improving cell-mediated immunity in infected udders.

The real implication of the influence on t_max_ by the rs426249393 SNP at *SPP1* is still to be clarified. The parameter t_max_ was much shorter in heterozygous AG, and we can speculate this was attributable to one of the many secondary and still unknown roles exerted by osteopontin, a multifaceted protein [17]. In fact, Sheehy et al. [15] reported that during lactation of dairy cows, the gene product of *SPP1* regulates the level of expression of two casein genes codifying for β and κ-casein, *CSN2* and *CSN3*, the latter being strictly involved in rennet coagulation of milk [44].

The *POFUT1* gene emerges as a positional candidate gene in relation to mammary gland development traits [11], and it also appears in the database of Ogorevc et al. [45] of cattle candidate genes for milk production. The expression of this gene is fundamental for the correct formation of the epithelial and myoepithelial cells that form the mammary alveolus [46]. We genotyped three intronic SNPs at *POFUT1*, one of which was monomorphic while two others were heterozygous and in high LD, forming a haplotype block. *POFUT1* SNP rs424501869 was associated with two different traits of curd firmness, a_45_ and a_60_. These two curd firmness traits have been measured in addition to the traditional value of a_30_, which is the curd firmness in mm that is observed 30 min after rennet addition, while a_45_ and a_60_ showed decreasing values and are linked to the phases of curd syneresis and whey expulsion [25]. The association of *POFUT1* with this particular phase of milk coagulation was also found for SNP rs408068827, showing association with t_max_. The t_max_ parameter is derived from the modeling of milk firmness [47] and represents the point at which CF_t_ attains its maximum level and the point at which the effects of the two parameters k_CF_ and k_SR_ are equal but opposite in sign [26]. To the best of our knowledge, an association between *POFUT1* polymorphisms and milk coagulation properties was highlighted in this investigation for the first time, although the underlying mechanisms still have to be elucidated. Genome-wide and pathway-based association, which are more informative than the analysis of a single or a limited number of SNPs, have revealed that milk coagulation and cheese-making traits in the bovine species are affected by the combined additive effect of clustered genes [48,49].

The *PRLR* gene encoding the prolactin receptor has emerged from genome wide studies because of its position in a genome region where positive selection was highlighted in sheep [11,20]. Here we investigated three intronic SNPs. Two of them had a high LD value, but none of the three were involved in a haplotype block. Only SNP rs400874750 had a significant association with the lactose and SCS content. The prolactin receptor has been mainly investigated as a regulator of reproduction, lactation and the association with milk traits in dairy cattle [20]. A stimulating finding from the present study showed by the polymorphism at rs400874750 was the unfavorable values recorded for concentration of lactose (lower) and SCS (higher) in milk samples from CC homozygote ewes (Figure 1f,h). Among the several processes occurring in the mammary epithelium, it is worth noting that macrophage activation and the synthesis of milk lactose are mediated by the PRL/PRLR system [21]. The effect of rs400874750 on milk coagulation traits, RCT and k_20_ (Figure 1h,i), may be explained by the link between the PRL/PRLR system and the synthesis of milk proteins. Indeed, some negative isoforms of PRLR expressed in vitro in bovine mammary cells inhibit the transcription of milk protein genes [21], and those may be consequently involved in the complex stages from milk to gel formation and cheese-making [44]. The results recorded in the present study are in accordance with what has been recorded for the sheep species, since the PRLR short form has a negative effect on the activation of milk protein gene transcription [50], and lactose and SCS are indirect markers of udder inflammation and inversely correlated in milk (the higher the SCS the lower the lactose, and vice versa), causing the delay of rennet coagulation and curd firming times [43]. 

## 5. Conclusions

The polymorphism of SNPs at *SPP1*, *POFUT1* and *PRLR* were investigated for the first time in the Sarda sheep, a specialized dairy breed. The present study evidenced a significant association between SNPs at the candidate genes and many milk traits in the sheep species. Despite the possibility that other SNPs that were not investigated in the present study might be responsible for the observed effects, the results represent encouragement to conduct further research and achieve the improvement in production from dairy sheep farms and cheesemaking plants.

## Figures and Tables

**Table 1:** Single nucleotide polymorphisms (SNPs) genotyped at the *SPP1*, *POFUT1* and *PRLR* genes in Sarda sheep (n = 380).

Genes and SNP ID	Chr pos.	Gene Region	ObsH	PredH	HWpv	%Gen	MAF	Alleles
*SPP1*								
rs161844011	OAR6:36651870	exon 7 ^1^	0.434	0.468	0.202	98.1	0.373	(T):C
rs426249393	OAR6:36658163	exon 1 ^2^	0.384	0.394	0.672	99.2	0.27	A:(G)
*POFUT1*								
rs424501869	OAR13:61007495	intron 1	0.446	0.476	0.266	100.0	0.39	A:(G)
rs421284407	OAR13:61009391	intron 2	0.0	0.0	1.0	93.3	0.0	C
rs408068827	OAR13:61013709	intron 3	0.422	0.454	0.206	100.0	0.348	A:(C)
*PRLR*								
rs412695065	OAR16:38969344	intron 1	0.402	0.370	0.334	100.0	0.245	A:(C)
rs400874750	OAR16:39004070	intron 2	0.538	0.488	0.223	100.0	0.421	T:(C)
rs428472303	OAR16:39006813	intron 2	0.506	0.467	0.3585	96.7	0.371	T:(C)

SNP ID: dbSNP reference records; Chr pos.: chromosome position on Oar_v3; OAR: *Ovis aries*; ObsH: observed heterozygosity; PredH: predicted heterozygosity; HWpv: Hardy–Weinberg test *p*-Value; %Gen: percentage of genotyped samples; MAF: minor allele frequency (minor allele in brackets); ^1^ p.Gln235Arg; ^2^ 5’ untranslated region.

## Supplementary Materials

The following are available online at https://www.mdpi.com/2076-2615/10/7/1216/s1, Table S1: Context sequences of the 8 SNPs investigated at SPP1, POFUT1 and PRLR in the population of the Sarda sheep breed. Table S2. Descriptive statistics of milk yield and composition, milk coagulation properties (MCP) and curd firmness over time traits (CFt) from the sampled population of Sarda sheep (n = 380); Table S3. F-value and significance for milk yield and composition, milk coagulation properties (MCP) and curd firmness over time traits (CFt) according to the effect of each of the 7 polymorphic SNPs out of the 8 investigated at ovine SPP1, POFUT1 and PRLR genes in Sarda sheep (n = 380); Table S4. F-value and significance for milk yield and composition, milk coagulation properties (MCP) and curd firmness over time traits (CFt) according to the effect of LD Block1 at POFUT1 gene in Sarda sheep (n = 380).
