# Supplementary material for: Association Analysis between SPP1, POFUT1 and PRLR Gene Variation and Milk Yield, Composition and Coagulation Traits in Sarda Sheep
Source: Animals (Basel). 2020 Jul 17;10(7):1216. doi: 10.3390/ani10071216 (PMC7401589; doi:10.3390/ani10071216)
Supplement: Supplementary file 1 [file animals-10-01216-s001.pdf]

Supplementary Information Table S1. Context sequences of the 8 SNPs investigated at *SPP1, POFUT1* and *PRLR* in the population of the Sarda sheep breed.

**Genes and SNP ID**	**Context Sequence**
SPP1	
rs161844011	TTCTTGGCTGAGTTTGGAATTTTCC[T/C]GACTNTCGATCNGATTGGAATGCTT
rs426249393	CTGCAGGCTTACCTTGGTCTGCAGC[A/G]GCAGAGAAGAGTCCAGTCCCCTGTG
POFUT1	
rs424501869	GCAGCCTGCACAATTCCCTAGCTGG[G/A]ATCACCCTCCTCTTGCCTCTGTGCC
rs421284407	ATGCCATTTTTAGAGAGATTTTTAA[C/A]AGAGGATAAAAGCCAGAGTAATGAG
rs408068827	CAAAGAGAGGTTCAATATTTTGCCC[A/C]AAATCATATAGCAAATAAGTTGTGG
PRLR	
rs412695065	CAAGTGAACCCTGAGGTAAGGGGAA[A/C]TTGACACGTGCCCCTCTGTCACACC
rs400874750	TCTCTTTCACTTCCTGGGTTATTTG[T/C]ACAAGAGGANGGAGAATCCATCCC
rs428472303	GAATGGTCACAGAGTTGAATGGACC[T/C]CCATATTGACTCCAGTACCTTCTTT

Supplementary Information Table S2. Descriptive statistics of milk yield and composition, milk coagulation properties (MCP) and curd firmness over time traits (CF_t) from the sampled population of Sarda sheep (n = 380).

**Milk traits**	**mean**	**SD**	**min**	**max**	**kurtosis**	**skewness**
Milk yield and composition						
dMY (g/day)	1641	899	183	5760	1.36	1.00
dFPY (g/day)	193	103	17	675	2.11	1.08
Fat (g/100mL)	6.50	1.23	3.86	12.52	1.75	0.77
Protein (g/100mL)	5.46	0.72	3.96	8.24	0.57	0.85
Casein (g/100mL)	4.27	0.61	2.99	6.67	0.68	0.86
Lactose (g/100mL)	4.81	0.28	2.70	5.5	7.93	-1.46
pH	6.66	0.09	6.45	7.12	1.97	0.69
SCS	4.72	2.18	0.16	10.68	0.07	0.62
LBC	2.54	0.93	0.60	4.23	-0.87	0.07
MCP						
RCT (min)	8.77	3.81	2.15	35.45	11.02	2.52
k_{20} (min)	1.93	0.54	1.30	7.00	22.62	3.15
a_{30} (mm)	50.28	11.49	4.14	70.00	0.08	-0.72
a_{45} (mm)	46.23	14.37	6.00	72.00	-0.67	-0.46
a_{60} (mm)	42.53	15.69	3.98	75.64	-0.80	-0.18
CF_t						
RCT_{eq} (min)	9.80	4.01	4.24	35.91	10.86	2.65
CF_P (mm)	685	3334	7	29024	42.38	6.29
k_{CF} (% × min^{-1})	0.28	0.13	0.01	0.90	2.36	0.74
k_{SR} (% × min^{-1})	0.014	0.018	0.001	0.114	11.21	3.09
CF_{max} (mm)	5449	918	5	7560	3.66	-0.98
t_{max} (min)	30.08	12.77	12.00	60.00	0.09	1.02

dMY: daily milk yield; dFPY: daily fat plus protein yield; SCS: somatic cell score = $\log_2 (SCC \times 10^{-5}) + 3$; LBC: logarithmic bacterial count = $\log_{10}$ total bacterial count (total bacterial count/1,000); RCT: rennet coagulation time; k_{20}: curd firming time; a_{30}, a_{45}, and a_{60}: curd firmness 30, 45 and 60 minutes after rennet addition; RCT_{eq}: rennet coagulation time estimated by the CF_t equation; CF_P: the maximum potential curd firmness after an infinite time; k_{CF}: curd-firming rate constant; k_{SR}: syneresis rate constant; CF_{max}: maximum curd firmness; and t_{max}: time to attain CFmax.

Supplementary Information Table S3. *F*-value and significance for milk yield and composition, milk coagulation properties (MCP) and curd firmness over time traits (CF_t) according to the effect of each of the 7 polymorphic SNPs out of the 8 investigated at ovine *SPP1*, *POFUT1* and *PRLR* genes in Sarda sheep (n = 380).

**Genes and**	**Milk yield and composition**									**MCP**					**CF_t**					
**SNP ID**	**dMY**	**dFPY**	**Fat**	**Protein**	**Casein**	**Lactose**	**pH**	**SCS**	**LBC**	**RCT**	**k_{20}**	**a_{30}**	**a_{45}**	**a60**	**RCT_{eq}**	**CF_P**	**k_{CF}**	**k_{SR}**	**CF_{max}**	**t_{max}**
SPP1																				
rs161844011	1.57	0.25	0.29	0.53	0.24	2.80	1.13	5.98**	1.04	0.38	0.51	1.07	0.70	1.70	0.44	1.02	0.10	1.05	1.56	2.21
rs426249393	0.80	2.15	1.36	0.32	0.34	0.62	0.05	1.25	0.07	0.19	0.69	0.10	0.37	0.45	0.55	0.57	0.97	2.45	0.12	3.14*
POFUT1																				
rs424501869	1.69	0.74	0.92	1.17	1.38	0.10	0.25	0.06	0.26	0.39	0.09	1.33	4.32*	5.03**	2.00	0.63	0.73	1.60	2.17	1.84
rs408068827	2.01	0.73	1.24	2.33	2.44	0.61	0.11	0.29	0.47	1.00	1.02	0.74	1.50	2.28	0.70	0.43	0.43	0.50	1.95	3.66*
PRLR																				
rs412695065	0.15	0.74	1.64	1.39	1.46	0.11	0.21	0.30	0.52	0.08	0.54	2.34	1.40	0.50	0.81	1.14	0.38	0.45	1.83	1.13
rs400874750	1.32	0.32	0.08	0.56	0.38	4.08*	0.33	0.04*	0.45	4.16*	3.14*	0.73	0.49	0.83	0.74	0.08	0.25	0.46	0.29	2.38
rs428472303	1.70	1.60	1.06	0.41	0.51	0.41	1.32	1.28	1.92	0.14	0.53	0.08	0.32	0.59	0.42	0.36	1.24	0.30	0.34	2.15

dMY: daily milk yield; dFPY: daily fat plus protein yield; SCS: somatic cell score = $\log_2$ (SCC × 10^{-5}) + 3; LBC: logarithmic bacterial count = $\log_{10}$ total bacterial count (total bacterial count/1,000); RCT: rennet coagulation time; k_{20}: curd firming time; a_{30}, a_{45}, and a_{60}: curd firmness 30, 45 and 60 minutes after rennet addition; RCT_{eq}: rennet coagulation time estimated by the CF_t equation; CF_P: the maximum potential curd firmness after an infinite time; k_{CF}: curd-firming rate constant; k_{SR}: syneresis rate constant; CF_{max}: maximum curd firmness; and t_{max}: time to attain CFmax.

** $P < 0.01$; * $P < 0.05$; no asterisk: non significant.

Supplementary Information Table S4. *F*-value and significance for milk yield and composition, milk coagulation properties (MCP) and curd firmness over time traits (CF_t) according to the effect of LD Block1 at *POFUT1* gene in Sarda sheep (n = 380).

	Milk yield and composition									MCP					CF_t					
	**dMY**	**dFPY**	**Fat**	**Protein**	**Casein**	**Lactose**	**pH**	**SCS**	**LBC**	**RCT**	k_{20}	a_{30}	a_{45}	**a60**	RCT_{eq}	CF_P	k_{CF}	k_{SR}	CF_{max}	t_{max}
LD Block1 at *POFUT1*	1,02	0,78	0,20	0,67	0,85	3,12*	1,58	0,82	0,00	5,12**	10,51***	6,02**	2,41	0,75	0.56	1,44	0,77	0,48	2,91	6,28**

dMY: daily milk yield; dFPY: daily fat plus protein yield; SCS: somatic cell score = $\log_2 (SCC \times 10^{-5}) + 3$; LBC: logarithmic bacterial count = $\log_{10}$ total bacterial count (total bacterial count/1,000); RCT: rennet coagulation time; k_{20}: curd firming time; a_{30}, a_{45}, and a_{60}: curd firmness 30, 45 and 60 minutes after rennet addition; RCT_{eq}: rennet coagulation time estimated by the CF_t equation; CF_P: the maximum potential curd firmness after an infinite time; k_{CF}: curd-firming rate constant; k_{SR}: syneresis rate constant; CF_{max}: maximum curd firmness; and t_{max}: time to attain CFmax.

*** $P < 0.001$; ** $P < 0.01$; * $P < 0.05$; no asterisk: non significant.
